# Ammonia Synthesis via Electrochemical Conversion

**DOI:** 10.3390/molecules31111805

**Published:** 2026-05-24

**Authors:** Jesús M. Martín-Marroquín, Dolores Hidalgo

**Affiliations:** Area of Circular Economy and Biotechnology, CARTIF Technology Centre, 47151 Boecillo, Valladolid, Spain; jesmar@cartif.es

**Keywords:** ammonia production, catalysts, electrocatalysis, electrolytes, nitrogen reduction reaction (NRR), nitrate reduction

## Abstract

Ammonia is a key chemical for fertilizers, industrial processes, and emerging energy applications, yet its conventional production via the Haber–Bosch process is associated with high energy demand and significant greenhouse gas emissions. In this context, electrochemical routes for ammonia synthesis have attracted increasing attention as a potential sustainable alternative, enabling nitrogen conversion under milder conditions and using renewable electricity. This review examines recent advances in electrochemical ammonia production, focusing on nitrogen reduction mechanisms, catalyst development, and electrochemical system design. The main reaction pathways for nitrogen activation are analyzed, together with the role of electrocatalysts in determining activity and selectivity. Progress in catalyst engineering, electrolyte optimization, and reactor configuration is discussed, with particular emphasis on strategies to mitigate competing reactions such as hydrogen evolution. In addition, alternative approaches based on nitrate reduction are considered due to their promising performance and potential integration with wastewater treatment. Unlike many recent reviews primarily focused on catalyst development or individual reaction pathways, this review provides an integrated perspective encompassing nitrogen reduction, nitrate reduction, electrolyte engineering, reactor architectures, and techno-economic considerations, thereby highlighting the interdependence between materials design, reaction environment, and system-level integration for scalable electrochemical ammonia synthesis.

## 1. Introduction

Ammonia is among the most extensively produced chemicals worldwide, with an annual output exceeding 180 million tonnes. Its main use lies in the production of nitrogen-based fertilizers, which are essential for sustaining the global food supply. In addition to agriculture, ammonia is widely applied in chemical synthesis, explosives manufacturing, pharmaceuticals, and refrigeration technologies. More recently, it has attracted renewed interest as an energy carrier due to its high hydrogen density, relatively straightforward liquefaction, and well-established global transport infrastructure. These features make ammonia a promising option for renewable energy storage and distribution in future low-carbon energy systems [1,2].

Despite its relevance, ammonia production continues to rely predominantly on the Haber–Bosch process, developed in the early twentieth century. This process involves the catalytic reaction of nitrogen and hydrogen at elevated temperatures (350–500 °C) and pressures (150–350 bar), typically using iron-based catalysts. While highly optimized and industrially mature, the Haber–Bosch process is associated with substantial energy consumption, accounting for approximately 1–2% of global energy use. Furthermore, its dependence on fossil-derived hydrogen (mainly produced via steam methane reforming) results in considerable carbon dioxide emissions [3]. These drawbacks have motivated increasing efforts to identify more sustainable alternatives for ammonia synthesis [4,5].

The transition toward renewable energy systems further reinforces the need for alternative ammonia production routes. Electricity generated from solar and wind sources is inherently variable, requiring effective energy storage solutions to ensure reliability and grid stability [6]. In this context, ammonia has emerged as a potential chemical storage medium, as it can be synthesized using renewable electricity, stored and transported in liquid form under moderate conditions, and subsequently utilized as a fuel or hydrogen carrier [7]. This perspective has stimulated growing interest in nitrogen fixation technologies capable of operating under milder and more sustainable conditions compared with the Haber–Bosch process [8].

Among the various alternatives, electrochemical ammonia synthesis has gained considerable attention. In these systems, nitrogen-containing species are reduced to ammonia using electrons supplied through an external circuit and protons typically derived from water. When powered by renewable electricity, such processes could enable near-carbon-neutral ammonia production [9]. Unlike the Haber–Bosch process, electrochemical routes can, in principle, operate under ambient temperature and pressure, offering opportunities for decentralized and flexible production schemes [10,11].

Electrochemical ammonia formation is most commonly associated with the nitrogen reduction reaction (NRR), where molecular nitrogen is converted to ammonia on the surface of an electrocatalyst. During this process, nitrogen molecules adsorb onto active sites and undergo successive proton-coupled electron transfer steps until ammonia is formed and released [12]. The overall reaction can be expressed as: N_2_ + 6H^+^ + 6e^−^ → 2NH_3_. Despite its apparent simplicity, this reaction is hindered by significant thermodynamic and kinetic barriers. The exceptional stability of the N≡N triple bond, with a dissociation energy of approximately 945 kJ mol^−1^, makes nitrogen activation particularly challenging under ambient conditions. Effective catalysts are therefore required to weaken this bond while facilitating subsequent hydrogenation steps [4].

A major limitation of electrochemical nitrogen reduction is the competition with the hydrogen evolution reaction (HER). In aqueous systems, protons and electrons tend to preferentially form hydrogen gas due to more favorable reaction kinetics and thermodynamics. This competition reduces ammonia selectivity and efficiency, often resulting in low Faradaic efficiencies and limited production rates [12]. Consequently, suppressing HER while promoting nitrogen activation remains a central challenge in the design of effective electrocatalysts.

To overcome these issues, extensive research has focused on developing advanced catalytic materials that enhance nitrogen adsorption and improve reaction selectivity [13]. A broad range of materials has been explored, including noble metals, transition metals, metal oxides, nitrides, and carbon-based catalysts [10,14,15]. Particular attention has recently been given to single-atom catalysts [16], bimetallic systems [17], and defect-engineered materials [18], which offer tunable active sites and enhanced catalytic performance. These approaches often rely on modifying the electronic structure through strategies such as heteroatom doping, vacancy creation, and surface nanostructuring to optimize nitrogen adsorption and reaction pathways [8]. More recently, oxide-based electrocatalysts and oxide–oxide heterostructures have also attracted increasing interest, particularly for nitrate reduction to ammonia. In these systems, oxide phase composition, interfacial electronic interactions, and metal–oxide coupling can effectively regulate nitrate adsorption, intermediate transfer, and NH_3_ selectivity, further expanding current catalyst design strategies for electrochemical ammonia synthesis [19,20].

Beyond catalyst design, the electrolyte and reaction environment also play a critical role in determining system performance [21]. Electrolyte properties influence proton availability, ion transport, and nitrogen solubility, thereby affecting both kinetics and selectivity. Additionally, the use of specific additives or tailored electrolyte compositions can help suppress hydrogen evolution and stabilize reaction intermediates. As a result, electrolyte engineering has become a key area of research for improving electrochemical ammonia synthesis [22].

In addition to direct nitrogen reduction, alternative electrochemical pathways have been explored. One notable example is nitrate reduction to ammonia [9,23]. This approach is particularly attractive because nitrates are commonly present in industrial effluents and agricultural runoff, contributing to environmental pollution. Their electrochemical conversion to ammonia offers a dual benefit: pollutant removal and resource recovery. Moreover, nitrate reduction generally exhibits more favorable kinetics than NRR due to the lower bond energy of nitrogen–oxygen bonds and higher solubility in aqueous media [4,24].

Another emerging strategy involves lithium-mediated nitrogen reduction. In this process, lithium reacts with nitrogen to form lithium nitride, which is subsequently protonated to produce ammonia [25]. Although this route has shown high selectivity in some studies, it requires highly reducing potentials and non-aqueous electrolytes, posing challenges in terms of energy efficiency and operational stability [26].

Despite significant progress, electrochemical ammonia synthesis is still at an early stage of development. Many systems reported to date exhibit low production rates and limited energy efficiency, hindering their scalability for industrial applications [27]. In addition, reliable ammonia detection remains a critical issue, as contamination and measurement inaccuracies can lead to overestimated performance. This has highlighted the importance of standardized protocols and rigorous experimental validation [1].

Nevertheless, ongoing advances in catalyst development, system design, and mechanistic understanding continue to improve the prospects of this technology. With further improvements in activity, selectivity, and energy efficiency, electrochemical nitrogen fixation could complement or partially replace conventional ammonia production, particularly in decentralized or renewable-based systems. Although numerous reviews on electrochemical ammonia synthesis have been published in recent years, many of them focus predominantly on specific aspects such as catalyst development, nitrogen reduction mechanisms, or lithium-mediated systems. Comparatively less attention has been devoted to integrating these approaches with electrolyte effects, reactor engineering, and techno-economic constraints within a unified framework. In addition, recent advances in electrochemical nitrate reduction have often been discussed separately from direct nitrogen reduction, despite their growing relevance as potentially more practical ammonia production routes.

In this context, the present review aims to provide a broader and more critical perspective by comparatively examining the interplay between catalytic materials, electrolyte environment, reactor configuration, and sustainability considerations across the main electrochemical ammonia synthesis pathways. This review therefore not only summarizes recent progress in electrochemical ammonia synthesis, but also emphasizes the relationships between mechanistic understanding, system-level design, and practical scalability. It first discusses the fundamental principles of nitrogen activation and reduction, followed by an analysis of reaction mechanisms. Advances in catalyst design, electrolyte optimization, and reactor configurations are then examined, along with alternative pathways such as nitrate reduction. Finally, key scientific and technological challenges are identified, together with future directions toward scalable and sustainable ammonia production.

## 2. Core Principles of Electrochemical Ammonia Synthesis

Electrochemical ammonia synthesis is governed by the complex interaction between surface reaction kinetics, interfacial phenomena, and mass transport at the electrode–electrolyte boundary. Together, these factors determine the achievable activity, selectivity, and overall energy efficiency of the process [28]. The conceptual basis of this process originates from the Haber–Bosch mechanism, where nitrogen and hydrogen adsorb on catalyst surfaces, followed by sequential hydrogenation to form ammonia. In thermocatalytic systems, nitrogen dissociation is typically the rate-limiting step due to the high activation energy required, which necessitates elevated temperatures. Under electrochemical conditions, however, direct dissociation of nitrogen at ambient conditions is highly unfavorable, and alternative pathways must be considered.

Mass transport represents an additional limitation. The low solubility of nitrogen in aqueous electrolytes (approximately 0.7 mmol L^−1^ at room temperature) restricts its availability at catalytic sites, limiting achievable current densities and ammonia production rates [6]. Even highly active catalysts may therefore operate under reactant-starved conditions. Approaches such as gas diffusion electrodes and operation under elevated pressure have been explored to improve nitrogen supply, although they introduce added system complexity and may alter local reaction environments.

A key challenge in electrochemical ammonia synthesis is the competition with the HER. In aqueous media, HER is both thermodynamically competitive and kinetically favored due to its simpler reaction pathway. Although the standard potentials of HER (0 V vs. RHE, where RHE refers to the reversible hydrogen electrode) and nitrogen reduction (≈−0.092 V vs. RHE) are relatively close, HER dominates electron consumption, significantly reducing ammonia selectivity [6]. As a consequence, many reported systems exhibit Faradaic efficiencies below 15% and ammonia production rates lower than 10^−10^ mol s^−1^ cm^−2^, far from practical targets [10].

System performance is typically assessed through several key indicators, including ammonia yield rate, Faradaic efficiency (FE), and energy efficiency. The latter is particularly relevant when considering integration with renewable energy sources [29]. For example, lithium-mediated systems can reach Faradaic efficiencies above 90%, but their overall energy efficiency is limited to around 28% due to the highly negative potentials required for lithium deposition [7]. This value remains well below both the ~60% target for electrochemical synthesis and the ~75% efficiency associated with Haber–Bosch.

The electrochemical environment also plays a decisive role. Electrolyte composition influences proton availability, ionic transport, and interfacial charge transfer [30]. While aqueous systems favor HER due to high proton concentration, non-aqueous or hybrid electrolytes can suppress proton activity and improve ammonia selectivity, albeit with trade-offs in conductivity, stability, and scalability [31]. Additionally, local variations in pH and reactant concentration at the electrode surface can further affect reaction pathways and intermediate stabilization [10].

Reliable ammonia quantification remains a major experimental challenge due to the extremely low production rates typically observed [32]. Trace contamination from reagents or experimental setups can lead to significant overestimation of catalytic performance. Therefore, rigorous validation methods, including isotopic labeling with ^15^N_2_ [33] and well-designed control experiments [34], are essential to ensure data reliability.

The performance of current electrochemical ammonia synthesis systems is constrained by a combination of intrinsic and practical limitations, including slow kinetics, high activation barriers, mass transport restrictions, and competition from HER. As a result, key performance targets such as current densities of ~300 mA cm^−2^, high selectivity, and long-term stability remain unmet [21]. Addressing these issues requires coordinated progress in catalyst design, electrolyte optimization, and reactor engineering.

The nature of the nitrogen source also significantly influences reaction behavior. In addition to direct nitrogen reduction, alternative pathways such as nitrate reduction have demonstrated improved kinetics and higher ammonia production rates. Electrochemical ammonia synthesis can therefore be broadly classified based on the nitrogen precursor, including molecular nitrogen (N_2_) and oxidized species such as nitrate (NO_3_^−^), each involving distinct mechanisms and limitations.

### 2.1. Reaction Mechanisms in Electrochemical Nitrogen Reduction

**Figure 1:**
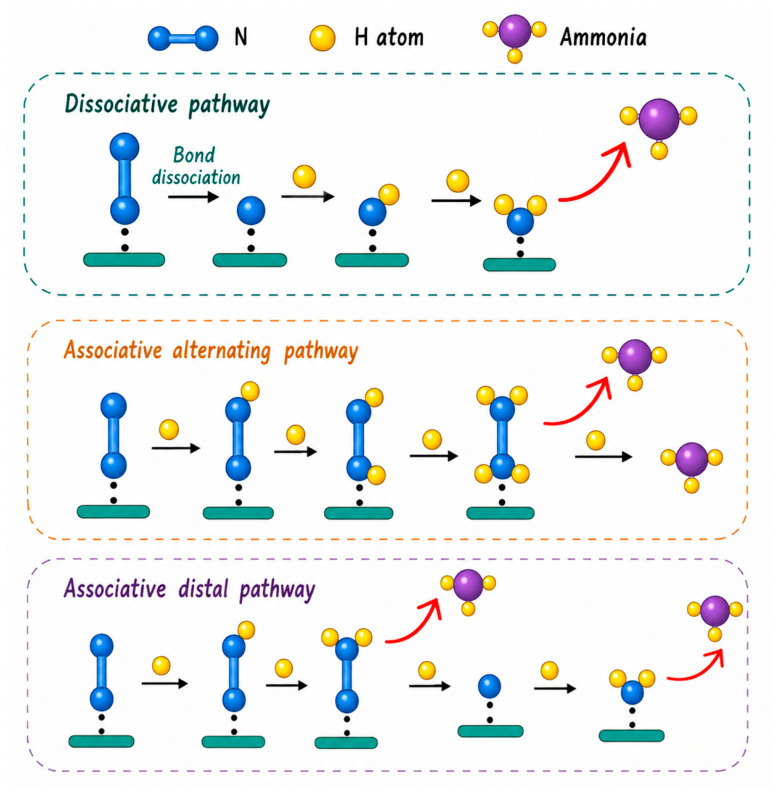
Schematic overview of the main mechanistic pathways involved in electrochemical nitrogen reduction to ammonia.

The main challenge in nitrogen reduction arises from the exceptional stability of the N≡N triple bond, making nitrogen one of the most inert molecules in nature. This translates into high activation barriers for the initial proton-coupled electron transfer step, often identified as the rate-determining step. Furthermore, nitrogen adsorption on catalyst surfaces is inherently weak due to its nonpolar character, requiring active sites capable of promoting charge transfer into antibonding orbitals. This difficulty is further compounded by nitrogen’s electronic properties, including a large energy gap (~10.8 eV), high ionization energy (~15.8 eV), and negative electron affinity (~−1.9 eV) [35]. Electrocatalytic nitrogen reduction can proceed through dissociative, associative, or enzymatic pathways [36], as illustrated in Figure 1. In the dissociative mechanism, nitrogen is first split into atomic species before hydrogenation, similar to the Haber–Bosch process [37]. However, this pathway is generally unfavorable under ambient electrochemical conditions due to the high energy required for bond cleavage.

More commonly, nitrogen reduction follows associative pathways, where hydrogenation begins before complete bond breaking [38,39]. In these mechanisms, nitrogen remains intact in the early stages, forming intermediates such as N_2_H, and the bond is gradually weakened as hydrogenation progresses. Computational studies have shown that associative routes can exhibit lower energy barriers than dissociative ones, depending on the catalyst surface [40].

Within associative mechanisms, different hydrogenation sequences can occur. In the distal pathway, one nitrogen atom is fully converted to ammonia before the second is reduced, whereas in the alternating pathway, both atoms are hydrogenated in parallel. The enzymatic pathway involves side-on adsorption of nitrogen, facilitating electron transfer into antibonding orbitals and enhancing activation [36]. Experimental detection of intermediates such as N_2_H, supports these mechanisms [41]. Nevertheless, a significant part of the current mechanistic understanding is still inferred from density functional theory (DFT) calculations combined with indirect experimental observations, since direct operando identification of short-lived intermediates under realistic electrochemical conditions remains highly challenging.

Ammonia formation proceeds through multiple proton-coupled electron transfer steps involving intermediates such as N_2_H*, NH*, and NH_2_* [1]. The stability of these intermediates is highly sensitive to catalyst properties, which explains the strong dependence of performance on electronic structure and surface configuration. The complexity of this six-electron process contributes to slow kinetics, particularly in the initial steps. An alternative mechanism, the Mars–van Krevelen pathway, has been proposed for transition metal nitrides [42]. In this case, lattice nitrogen participates directly in ammonia formation, generating vacancies that are subsequently replenished by gaseous nitrogen [43]. This mechanism can reduce competition with HER and improve selectivity [44].

### 2.2. Ammonia Synthesis via Electrochemical Nitrate Reduction

Nitrate reduction has attracted increasing attention due to both its environmental relevance and its favorable reaction characteristics. Approximately 26 million tons of nitrate are released annually into water bodies, representing nearly 19% of global fertilizer production [45]. This contributes to eutrophication and poses risks to human health [46]. Electrochemical nitrate reduction therefore offers the dual benefit of pollutant removal and ammonia production.

Compared with nitrogen reduction, nitrate activation is facilitated by the lower bond dissociation energy of N–O bonds (~204 kJ mol^−1^) and higher solubility in aqueous media, resulting in improved mass transport and faster kinetics. Under optimized conditions, ammonia production rates can reach the mmol cm^−2^ h^−1^ range, significantly higher than those typically obtained for NRR. However, controlling selectivity remains challenging due to the complex reaction network and the formation of byproducts such as N_2_ and NO_2_^−^ [4,24]. This difficulty arises from the multiple proton-coupled electron transfer steps involved in nitrate reduction, where reaction intermediates such as NO_2_, NO, and N_2_O* may follow competing pathways depending on catalyst properties, local proton availability, applied potential, and interfacial reaction environment. Excessive hydrogen adsorption may further promote side reactions or HER, reducing ammonia selectivity. Consequently, achieving high NH_3_ selectivity requires precise control over intermediate adsorption energies, hydrogenation kinetics, and local catalytic microenvironment.

The complexity of nitrate reduction arises from the wide range of nitrogen oxidation states involved, from +5 in NO_3_^−^ to −3 in NH_3_, leading to multiple intermediates including NO_2_^−^, NO, N_2_O, and N_2_ [47]. Achieving high ammonia selectivity requires suppressing competing pathways leading to undesired products [48]. Mechanistically, nitrate reduction can proceed via direct or indirect pathways [11]. The dominant pathway depends strongly on operating conditions such as nitrate concentration and pH. At high nitrate concentrations and strongly acidic conditions, indirect pathways involving reactive intermediates (e.g., NO^+^) become dominant [49,50]. In contrast, under more typical conditions, the direct pathway prevails [51].

**Figure 2:**
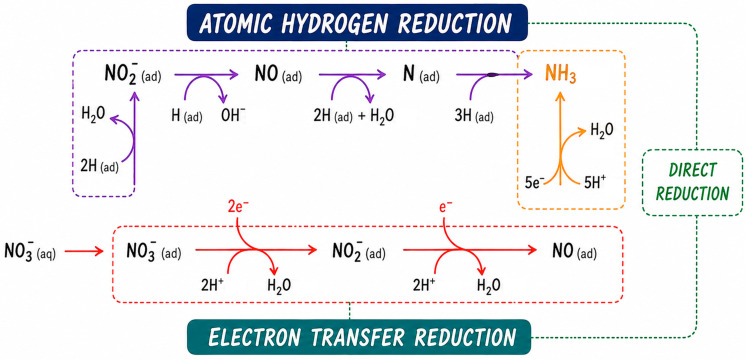
Schematic representation of the direct electrochemical nitrate reduction pathway toward ammonia.

In the direct mechanism, nitrate is adsorbed on the catalyst surface and reduced through a series of intermediates [52]. As illustrated in Figure 2, this route may proceed through two main pathways: a direct electron-transfer-mediated pathway and an adsorbed hydrogen-mediated pathway, usually denoted as H(ad)-mediated reduction [53]. In the electron-transfer route, the conversion of nitrate to nitrite is often the rate-determining step [54], followed by further reduction to NO, which plays a key role in determining product distribution [55]. Depending on its fate, NO can lead to ammonia or to gaseous byproducts such as N_2_O and N_2_ [56,57].

In parallel, the H(ad)-mediated pathway involves hydrogen species generated via the Volmer step [58], which promote hydrogenation of intermediates and favor ammonia formation [59,60]. However, excessive hydrogen production can reduce efficiency. Tailoring the local catalytic environment, for example through hydroxyl-rich interfaces, has been shown to enhance selectivity, achieving near-unity FE in some cases [61].

Catalyst properties, including electronic structure and active site configuration, are critical in determining performance. For instance, single-atom catalysts on nitrogen-doped carbon have demonstrated strong correlations between electron transfer capability and catalytic activity [62]. Specific coordination environments, such as Cu–N_4_ sites, can suppress undesired reaction pathways. Density functional theory studies have provided further insights into reaction energetics and intermediate stabilization [32].

Experimental validation of mechanisms is typically achieved using isotope labeling (^15^NO_3_^−^) combined with spectroscopic techniques [50,63]. In addition, operando methods such as Raman spectroscopy, FTIR, and electrochemical mass spectrometry enable real-time detection of intermediates, providing valuable information for catalyst design and mechanistic understanding. These studies demonstrate that ammonia selectivity in nitrate reduction depends not only on catalyst composition, but also on the dynamic interplay between intermediate stabilization, hydrogen availability, and interfacial reaction conditions.

## 3. Materials and Design Strategies for Electrochemical Ammonia Production

The performance of electrochemical ammonia synthesis is not defined by a single component, but by the combined behaviour of the catalyst, electrolyte, and reactor architecture. Although the preceding section addressed the main mechanistic routes involved in ammonia formation, the practical efficiency of these systems depends on how these elements are selected, engineered, and integrated. The electrocatalyst determines the adsorption, activation, and transformation of nitrogen-containing species; the electrolyte defines the local interfacial environment, proton availability, and ionic transport; and the reactor configuration controls reactant delivery, mass transfer, ohmic losses, product separation, and scalability. Accordingly, this section reviews the principal material and design factors governing electrochemical ammonia production, with particular attention to electrocatalyst development, electrolyte engineering, and electrolyzer configuration.

### 3.1. Electrocatalyst Design for Nitrogen-to-Ammonia Conversion

Efficient electrocatalysts are essential for improving both the NRR and electrochemical nitrate reduction toward ammonia. In NRR, the catalyst must promote the activation of highly stable N_2_ while simultaneously limiting the competing HER, which commonly dominates in aqueous media and keeps FE below 15% in many reported systems [10]. Nitrate reduction, by contrast, benefits from higher reactant solubility and more favourable reaction kinetics, allowing markedly higher ammonia production rates. However, it also requires strict control over adsorbed intermediates to avoid side pathways leading to products such as N_2_ and N_2_O [4,24].

In both routes, catalytic behaviour is largely dictated by the interaction between active sites and reaction intermediates. For NRR, the adsorption strength of N_2_ and partially hydrogenated species such as N_2_H* and NH* determines whether activation and hydrogenation can proceed efficiently without excessive surface poisoning. In nitrate reduction, the adsorption, stabilization, and further transformation of NO_2_^−^, NO, and related intermediates control whether the reaction proceeds selectively toward ammonia or diverges toward gaseous nitrogen products. For this reason, current catalyst design increasingly relies on tuning electronic structure, local coordination, surface defects, and interfacial properties so that adsorption energies and reaction pathways can be optimized [13]. Recent reviews have further highlighted that advanced nanomaterial engineering strategies, including heterostructure construction, defect modulation, and interface engineering, play a decisive role in improving nitrate adsorption, intermediate stabilization, and ammonia selectivity during electrochemical nitrate reduction [64].

**Figure 3:**
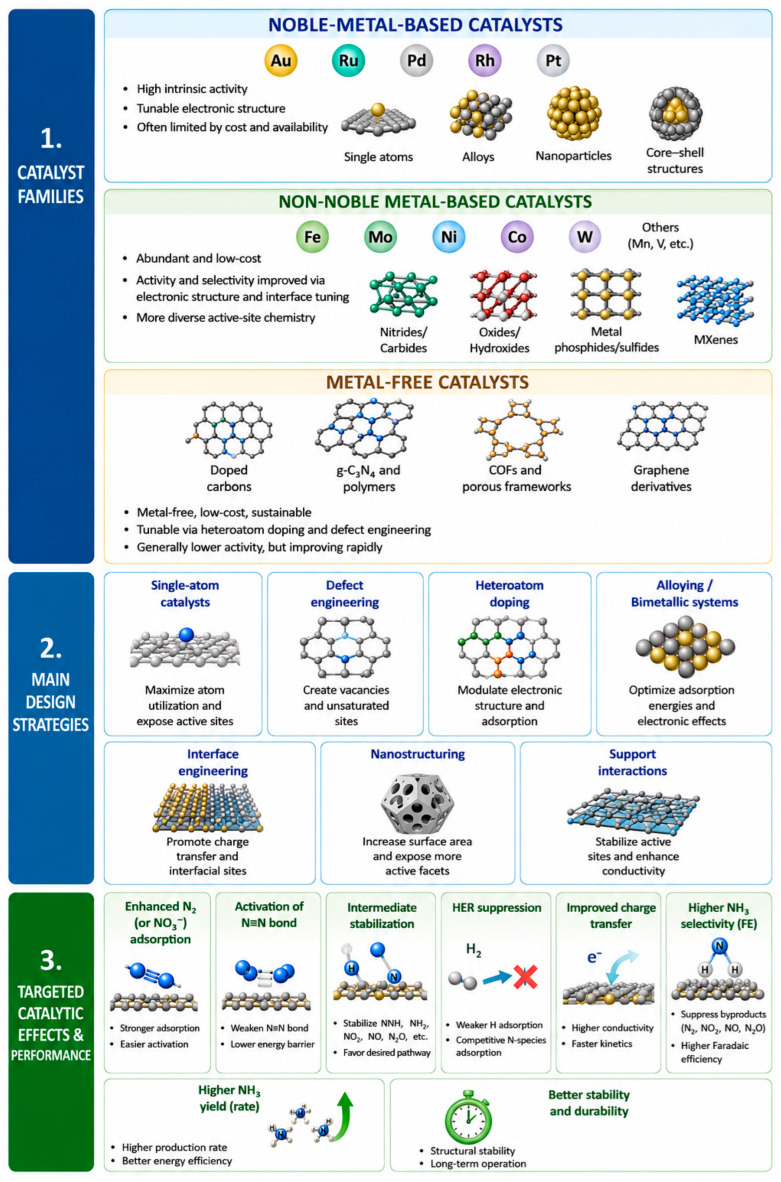
Classification of electrocatalysts and principal design strategies for electrochemical ammonia synthesis.

Particular attention has recently been devoted to oxide-based electrocatalysts and oxide phase engineering strategies for nitrate electroreduction. Recent studies have demonstrated that oxide phase composition, dual-phase nanodomains, and oxide–oxide heterointerfaces can strongly regulate nitrate adsorption, intermediate transfer, charge transport, and ammonia selectivity. In-plane integration of α/γ-Fe_2_O_3_ dual-phase nanodomains has been shown to enhance NO_3_^−^RR performance through the synergistic combination of highly active α-Fe_2_O_3_ sites and conductive γ-Fe_2_O_3_ domains, which promote electron transport while suppressing competitive hydrogen evolution pathways [19]. Similarly, Fe_2_O_3_-decorated Cu_2_O nanowires with abundant Cu–Fe interfacial sites have demonstrated improved nitrate reduction performance, where Fe_2_O_3_ facilitates nitrate adsorption and early-stage reduction, while Cu_2_O promotes intermediate hydrogenation and NH_3_ desorption, resulting in enhanced Faradaic efficiency and ammonia selectivity [20]. Recent reviews have additionally highlighted the relevance of oxygen-vacancy engineering, dynamic surface reconstruction, and defect-rich oxide nanostructures as emerging approaches for enhancing nitrate activation and optimizing ammonia selectivity during electrochemical nitrate reduction [64]. These findings further highlight the critical role of interfacial electronic coupling and oxide phase engineering in the rational design of high-performance electrocatalysts for sustainable nitrate-to-ammonia conversion. A diverse set of catalytic materials has been reported, including noble metals, transition metals, metal compounds, and metal-free carbon-based systems. Figure 3 summarizes the main catalyst families and the principal design strategies currently employed to enhance electrochemical ammonia synthesis performance, highlighting the relationships between active-site engineering, HER suppression, intermediate stabilization, and catalytic activity/selectivity. Table 1 summarizes representative electrocatalysts reported for electrochemical NRR toward ammonia synthesis, including ammonia yield, FE, and operating potential. In parallel, Table 2 presents representative electrocatalysts reported for the electrochemical nitrate reduction reaction (NO_3_^−^RR), reflecting the growing relevance of this alternative pathway for ammonia production. Both tables also reflect the wide variability in experimental conditions, including electrolyte composition, catalyst loading, reactor configuration, and nitrogen source or nitrate concentration across different studies. Therefore, the reported values should be interpreted as representative performance indicators rather than directly standardized benchmarks. The following subsections discuss the main catalyst families, emphasizing the relationships between structure, activity, and selectivity in both NRR and nitrate reduction.

#### 3.1.1. Noble Metal-Based Catalysts

Noble metal electrocatalysts, including Pt, Pd, Au, Ru, and Rh, have been widely examined for electrochemical ammonia synthesis because their electronic structures can favour the adsorption and activation of nitrogen-containing species [132]. In NRR, these catalysts may show high intrinsic activity and, in selected cases, notable ammonia yields and FE. Atomically dispersed noble metals and single-atom catalysts are particularly attractive because they maximize metal utilization while providing well-defined catalytic sites [71,133]. Nevertheless, their broader implementation is constrained by cost, limited abundance, and possible degradation under electrochemical operation [134]. Consequently, recent work has focused on improving catalytic efficiency through strategies such as single-atom dispersion, alloy formation, heteroatom doping, and interfacial engineering.

Among noble metals, Ru-based catalysts have received special attention owing to their strong N_2_ adsorption ability and adjustable hydrogen-binding characteristics [72,135,136]. Their activity is highly sensitive to nanoscale structure, with particle sizes close to 2 nm often providing favourable performance. For instance, Ru nanoparticles of approximately 1.9 nm supported on reduced graphene oxide (rGO) produced NH_3_ at 9.14 μg·h^−1^·mg_cat_^−1^ with an FE of 2.1% [72]. A higher activity was reported for Ru_2_P nanoparticles supported on rGO, which reached 32.8 μg·h^−1^·mg_cat_^−1^ and 13.04% FE at −0.05 V vs. RHE [73]. Mechanistic analysis identified the first hydrogenation step from N_2_ to N_2_H as the potential-determining step, with an energy barrier of around 0.68 eV, while electron transfer from the support was found to facilitate N_2_ activation [72]. Defect engineering can further enhance this behaviour. In Ru/2H-MoS_2_ systems, sulfur vacancies generate synergistic effects between Ru sites and the support, leading to ammonia production rates of 1.14 × 10^−10^ mol cm^−2^ s^−1^ and FE of 17.6%, while also suppressing HER [35].

Heteroatom doping offers another route to tune the electronic structure of noble metal catalysts. A mesoporous Rh catalyst co-doped with boron and sulfur (B,S-mRh/NF), for example, showed improved N_2_ adsorption and more selective hydrogenation toward ammonia, which was attributed to changes in the electronic density of Rh active sites [70].

Au-based catalysts are especially appealing because their intrinsically weak hydrogen adsorption can help inhibit HER and improve selectivity [137]. Carbon-supported Au nanoparticles of around 8 nm delivered NH_3_ yields of 17.49 μg·h^−1^·mg_cat_^−1^ with an FE of 5.79% [69]. More elaborate architectures have achieved substantially higher values. Hydrophobic organosilica-coated Au@MOF catalysts, for instance, reached NH_3_ yields up to 49.5 μg·h^−1^·mg_cat_^−1^ and FE as high as 60.9% at −0.3 V vs. RHE [138]. These improvements were associated with electronic modulation of Au sites together with restricted proton access at the interface. Au-based composites such as Au–BOx have also been reported to enhance N_2_ activation through charge transfer, achieving FE values above 50% [139].

Alloy engineering is another important strategy for improving noble metal performance. Bimetallic systems such as Au–Cu, Au_3_Cu, and PdCu can enhance N_2_ adsorption and activation through synergistic electronic interactions between the two metals [66,67,74]. These interactions tune the adsorption strength of key intermediates and can provide higher activity than the corresponding monometallic catalysts.

Pd-based catalysts further illustrate the importance of controlling electronic structure and hydrogen interactions. Palladium hydride nanorods (PdH_0.43_) achieved NH_3_ formation rates of 17.53 μg·h^−1^·mg_cat_^−1^ with an FE of 18.56%, outperforming pristine Pd because of modified hydrogen adsorption and improved hydrogenation kinetics [75]. Likewise, phosphidation approaches such as PdP_2_-rGO increased ammonia yield to approximately 30.3 μg·h^−1^·mg_cat_^−1^ with FE close to 12.6% by tuning the electronic structure of Pd sites [76].

Beyond alloying and doping, the interface between noble metals and their supports is also critical. Coupling noble metals with oxide or carbon supports can promote charge transfer and adjust the adsorption geometry of nitrogen-containing intermediates, thereby enhancing catalytic activity [65,140].

#### 3.1.2. Non-Noble Metal-Based Catalysts

Non-noble-metal catalysts have attracted increasing attention as lower-cost and more abundant alternatives for electrochemical ammonia synthesis [83,141]. Compared with noble metals, these materials often bind nitrogen-containing species more strongly. This can assist N_2_ activation, but excessive adsorption may slow subsequent hydrogenation or hinder ammonia desorption. Therefore, the design of non-noble catalysts commonly focuses on balancing adsorption strength, increasing defect density, and constructing interfaces that improve activity without sacrificing selectivity.

Fe-based catalysts are among the most intensively studied because of the central role of iron in Haber–Bosch catalysis and its intrinsic capacity to activate nitrogen [83]. In electrochemical systems, however, their performance depends strongly on morphology, oxidation state, and surface structure. Tranchida et al. [81] showed that Fe nanoparticles deposited on carbon cloth are highly sensitive to precursor concentration. Increasing FeCl_3_ concentration from 1 mM to 10 mM caused particle agglomeration and pore blockage, resulting in an exponential decrease in ammonia yield. At 1 mM FeCl_3_, the catalyst produced 5.5 μg·h^−1^·mg_cat_^−1^ with 3.8% FE at −0.35 V vs. RHE. After electrochemical activation, performance increased to 26.44 μg·h^−1^·mg_cat_^−1^ and 20.4% FE, which was attributed to oxygen vacancy formation and optimization of the Fe^2+^/Fe^3+^ ratio. Similarly, porous Fe-containing iron oxyhydroxide nanosheets reached 28.5 μg·h^−1^·mg_cat_^−1^ and 13.2% FE at −0.4 V vs. RHE, compared with 12.8 μg·h^−1^·mg_cat_^−1^ and 4.2% FE for bulk materials, confirming the importance of nanostructuring and active-site accessibility [82,94].

Fe doping is also effective because it modifies both electronic structure and defect concentration. Fe-doped TiO_2_ nanoparticles delivered 25.47 μg·h^−1^·mg_cat_^−1^ and 25.6% FE at −0.4 V vs. RHE in 0.5 M LiClO_4_, compared with 5.36 μg·h^−1^·mg_cat_^−1^ and 2.65% FE for pristine TiO_2_. This improvement was linked to a higher density of oxygen vacancies, which promoted N_2_ adsorption and activation [82]. Even stronger enhancement was observed for Fe-doped Bi_2_MoO_6_ nanosheets, which achieved 71.01 μg·h^−1^·mg_cat_^−1^ and 80.12% FE at −0.1 V vs. RHE, greatly exceeding the undoped material (14.05 μg·h^−1^·mg_cat_^−1^, 4.21% FE) [83]. In that system, Fe doping modulated the Lewis acidity of Bi sites, improving N_2_ adsorption and activation while lowering the reaction barrier. These examples show that Fe may act either as a catalytic centre or as an electronic promoter, depending on how it is incorporated.

Mo-based materials form another important family of non-noble catalysts due to their strong interaction with nitrogen species and their ability to form diverse compounds, including oxides, carbides, nitrides, and sulfides. Their activity is frequently improved through heterostructure construction and defect engineering, which can simultaneously favour N_2_ activation and suppress HER. Mo-doped SnO_2_/C catalysts, for example, achieved 24.03 μg·h^−1^·mg_cat_^−1^ and 7.11% FE, corresponding to 1.81- and 1.70-fold increases over pure SnO_2_. This enhancement was attributed to the combined effects of Mo doping, tubular morphology, and a hydrophobic carbon coating that restricted proton transport and reduced HER [90].

More complex Mo-based catalysts use heterointerfaces to separate different catalytic functions. Ni-doped MoO_2_ catalysts produced 10.6 μg·h^−1^·mg_cat_^−1^ at −0.5 V vs. RHE and 19.6% FE at −0.1 V, with Ni^3+^ species promoting defect formation, improving charge transfer, and accelerating reaction kinetics [91]. A representative case is the Mo_2_C–MoO_2_ heterostructure supported on rGO, which achieved 13.94 ± 0.39 μg·h^−1^·mg_cat_^−1^ and 12.72 ± 0.58% FE [88]. Density functional theory calculations showed that Mo_2_C adsorbs N_2_ more strongly (−1.47 eV) than MoO_2_ (−0.81 eV) and elongates the N–N bond from 1.114 Å to 1.254 Å, indicating efficient activation. In parallel, MoO_2_ presents a higher hydrogen adsorption barrier (ΔG_H* = 0.61 eV, compared with 0.24 eV for Mo_2_C), thereby suppressing HER. This example illustrates how separating N_2_ activation and HER inhibition across different interfacial domains can improve overall performance.

Defect-rich sulfide catalysts reinforce this concept. W-doped MoS_2_ enriched with sulfur vacancies achieved 62.42 μg·h^−1^·cm_cat_^−2^ and 22.34% FE at −0.5 V vs. RHE. The combined contribution of tungsten dopants and sulfur vacancies enhanced N_2_ adsorption, lowered hydrogenation barriers, and suppressed HER [142]. Mo_2_C–Mo_2_N heterostructures also showed notable activity, reaching 9.6 μg·h^−1^·cm^−2^ and 10.15% FE. Mechanistic studies indicated that Mo_2_C follows an associative hydrogenation pathway, whereas Mo_2_N operates through a Mars–van Krevelen mechanism involving lattice nitrogen, allowing complementary catalytic roles within the same material [143].

Multi-metal catalysts demonstrate how synergistic interactions can further improve activity and selectivity. Ni/Co layered double hydroxides delivered 52.8 μg·h^−1^·mg_cat_^−1^ and 11.5% FE at −0.7 V vs. RHE. Operando Raman spectroscopy showed that N_2_ is preferentially adsorbed at Co sites and subsequently hydrogenated at Ni sites, suggesting a site-specific division of catalytic functions [95]. Fe/Mo bimetallic MoS_2_/MoO_2_@Fe_2_O_3_ composites exhibited 16.5% FE and retained more than 90% of their activity after 15 h, owing to an increased electrochemically active surface area and optimized hydrophobicity [87]. Even higher activity was reported for Bi-doped CuFe catalysts, which reached 216.1 μg·h^−1^·cm_cat_^−2^ and 46.8% FE at −0.4 V vs. RHE. In this case, Bi doping suppressed water adsorption and HER while promoting the formation and hydrogenation of nitrogen intermediates [144].

#### 3.1.3. Carbon-Based and Metal-Free Catalysts

Metal-free electrocatalysts provide a distinct route for electrochemical ammonia synthesis, relying on electronic structure modulation rather than metal-centred active sites. In these materials, catalytic behaviour arises from charge redistribution within the lattice, creating active sites able to adsorb and activate N_2_ while limiting hydrogen adsorption and suppressing HER. Carbon-based materials have been especially widely studied because of their structural flexibility and the possibility of tailoring their electronic properties through heteroatom doping and defect engineering [145,146].

Heteroatom doping is one of the most effective strategies for enhancing the activity of carbon-based catalysts. The introduction of N, S, B, P, or halogen atoms produces charge polarization in the carbon framework, generating sites suitable for N_2_ adsorption. For example, Cl-doped reduced graphene oxide (Cl–rGO) achieved ammonia yields of 70.9 μg·h^−1^·mg_cat_^−1^ with an FE of 5.97% at −0.3 V vs. RHE. DFT calculations indicated that chlorine-induced electron redistribution facilitates N_2_ activation through an associative pathway [103]. Sulfur-doped graphene reached ammonia yields of 28.56 μg·h^−1^·mg_cat_^−1^ and FE values of 7.07%, with sulfur atoms acting as preferred adsorption centres while the graphene matrix supported efficient electron transfer [104]. More broadly, multi-heteroatom doping can generate synergistic effects that further adjust adsorption strength and reaction energetics [147,148,149].

Nitrogen-doped carbon materials constitute another important subgroup. Pyridinic and pyrrolic nitrogen species can act as active centres for N_2_ chemisorption and activation. By controlling both nitrogen content and speciation, ammonia production rates up to 1.40 mmol·g^−1^·h^−1^ have been reported [105]. Nitrogen doping also improves electronic conductivity and facilitates charge transfer during the reaction, in addition to creating adsorption sites [150]. Other doped porous carbons, including F-doped and B-doped materials, have shown improved N_2_ adsorption and catalytic stability, demonstrating the combined importance of electronic and structural optimization [102,151].

Boron-based systems activate nitrogen through a different principle. Because boron is electron-deficient, B atoms can behave as Lewis acid sites, strengthening interaction with N_2_ and promoting charge transfer into antibonding orbitals. Boron-doped graphene with BC_3_ configurations has been reported to lower activation barriers and improve catalytic performance [106]. Boron carbon nitride (BCN) materials allow Lewis acidity to be tuned through the B/N ratio, with boron-rich compositions showing higher ammonia formation rates because of reduced barriers for hydrogenation steps [107]. These observations agree with theoretical studies indicating that boron-containing materials can activate N_2_ efficiently even without metal active centres [152,153].

Other metal-free catalysts have also been investigated, including boron nitride (BN), graphitic carbon nitride (g-C_3_N_4_), black phosphorus, and covalent organic frameworks (COFs). Mesoporous BN provides higher surface area and more accessible active sites than bulk BN, resulting in improved ammonia yields [154]. Black phosphorus nanosheets exhibit enhanced activity at edge sites, where favourable adsorption geometries facilitate N_2_ reduction [112]. In COF-based materials, boron-rich linkages act as Lewis acid sites for N_2_ adsorption, although the low intrinsic conductivity of these frameworks generally requires coupling with conductive supports to improve electron transfer [109]. Hybrid metal-free systems, such as BN quantum dots/g-C_3_N_4_ heterostructures, further show that interfacial engineering can promote charge transfer and enhance catalytic activity [101].

### 3.2. Electrolyte Engineering and Interfacial Control

Electrolyte engineering is a central aspect of electrochemical ammonia synthesis because the electrolyte defines the reaction environment at the electrode–electrolyte interface. Beyond providing ionic conductivity, the electrolyte regulates proton availability, nitrogen transport, counterion effects, and interfacial charge transfer, thereby directly affecting reaction kinetics, selectivity, and FE. In NRR systems, electrolyte selection is especially important because it determines the balance between nitrogen protonation and HER, while also influencing N_2_ solubility and transport toward the catalyst surface [155]. Recent studies further show that electrolyte composition directly modulates the electrode–electrolyte interphase (EEI), stabilizing reaction intermediates and altering the local atomic-scale reaction environment [31].

pH is one of the key variables controlling electrolyte performance. Aqueous media are generally grouped into acidic electrolytes, such as HCl and H_2_SO_4_; alkaline electrolytes, such as KOH and NaOH; and neutral electrolytes, such as phosphate-buffered saline (PBS) and Na_2_SO_4_. Each provides a different balance between proton supply, conductivity, and HER activity. Acidic electrolytes offer abundant protons and thus facilitate proton-coupled electron transfer, but they also strongly promote HER, often limiting NRR selectivity. For example, MoS_2_-based catalysts in 0.1 M HCl exhibited extremely low FE of approximately 0.096%, mainly because hydrogen evolution dominated the cathodic process [156]. However, catalyst engineering can partly offset this limitation. Liang et al. showed that F-doped MoS_2_ in 0.05 M H_2_SO_4_ reached an FE of approximately 20.6%, owing to strain-induced defects that suppressed HER and improved N_2_ activation [157].

Alkaline electrolytes generally suppress HER more effectively by lowering proton activity, making them common in NRR studies. Nevertheless, their performance depends not only on pH but also on cation identity. KOH, for example, has been reported to outperform NaOH for Fe-based catalysts, producing higher ammonia rates due to more favourable K^+^ transport and interfacial kinetics [158]. This highlights that ion-specific effects can be as important as bulk proton concentration in determining catalytic behaviour.

Neutral electrolytes often provide a favourable compromise, limiting HER while maintaining sufficient proton availability for nitrogen hydrogenation. Wang et al. compared acidic, neutral, and alkaline media and found that, although acidic and alkaline electrolytes produced higher current densities, neutral PBS delivered higher FE (~2.4%) and ammonia formation rate (~4.9 μg·h^−1^·mg^−1^) [110]. This behaviour was attributed to slower HER kinetics in neutral media due to increased mass- and charge-transfer resistance, which reduces proton availability near the catalyst surface. Similarly, Wei et al. reported HER suppression following the order PBS > K_2_SO_4_ > KOH > H_2_SO_4_, confirming the advantage of buffered neutral systems [159]. These results show that proton availability must be carefully balanced: it should be sufficient for nitrogen hydrogenation, but not so high that HER dominates.

Despite their practical advantages, aqueous electrolytes have inherent limitations. Low N_2_ solubility, approximately 0.7 mmol·L^−1^, and high water activity restrict FE and ammonia production rates [155]. This has repeatedly been identified as a central bottleneck for scaling NRR systems and requires simultaneous optimization of electrolyte formulation and reactor design to improve mass transfer [160]. Gas diffusion electrodes and hydrophobic layers, including PTFE-coated gas diffusion layers, have therefore been introduced to enhance N_2_ delivery while limiting water access to catalytic sites [159].

Electrolyte additives provide another means of tuning interfacial properties. Inspired by molecular crowding in biological media, polymeric additives such as polyethylene glycol (PEG400) can decrease water activity and proton mobility through hydrogen-bonding interactions. Guo et al. showed that approximately 20 wt% PEG400 increased FE to around 32.13% in acidic electrolytes by suppressing HER, although this improvement was accompanied by higher solution resistance and lower ionic conductivity [161,162]. This indicates that structuring the electrolyte can substantially alter reaction pathways even when bulk conductivity decreases. More recent work confirms that strategies such as water-in-salt electrolytes and confined electrolyte environments can also reduce proton activity while preserving sufficient ionic transport, emphasizing the importance of decoupling proton availability from conductivity in NRR systems [163].

Counterions also play an important role. Alkali metal cations influence the electric double layer and reactant adsorption. Song et al. [147] found that ammonia production rates follow the order Li^+^ > Na^+^ > K^+^, which was attributed to the compact Stern layer formed by Li^+^ ions. This layer promotes N_2_ adsorption and electrostatically repels protons from the electrode surface. Recent studies further demonstrate that cation-dependent interfacial electric fields and solvation structures are decisive for stabilizing key intermediates, reinforcing the need to consider electrolyte–catalyst coupling effects [8]. These observations collectively highlight that electrolyte effects arise from a complex combination of proton availability, ion-specific interactions, and interfacial electric-field regulation rather than from pH alone.

Nonaqueous electrolytes have been widely investigated to overcome the limitations of water-based systems. They generally provide higher N_2_ solubility and lower proton activity, which can strongly suppress HER. Ionic liquid electrolytes, for example, have reached FE values of up to approximately 60%, while fluorinated aprotic systems have achieved around 32% [164,165]. Recent studies confirm that nonaqueous systems often offer higher selectivity because of reduced proton availability, although they remain constrained by low current densities and slow proton transport [166].

A particularly relevant nonaqueous route is lithium-mediated electrochemical ammonia synthesis (LiMEAS). In this process, lithium is electrochemically deposited and reacts with N_2_ to form Li_3_N, which is subsequently protonated to NH_3_ [167]. Lazouski et al. [168] showed that LiBF_4_ in tetrahydrofuran (THF), with ethanol as a proton source, enables efficient ammonia production, although the process is highly sensitive to water contamination because water promotes HER. Chang et al. [169] emphasized that the formation of a catalytic solid–electrolyte interphase (SEI) is central to selectivity and kinetics in lithium-mediated systems, with electrolyte composition controlling SEI structure and performance. Other studies showed that THF provides better stability than solvents such as diglyme or dimethoxyethane [170], while alternative proton donors, including tetraalkyl phosphonium salts [171] or hydrogen oxidation reaction (HOR)-derived protons [172], can further improve system performance. Taken together, these results show that electrolyte engineering is not merely a supporting aspect of electrochemical ammonia synthesis, but a central strategy for regulating proton activity, interfacial chemistry, nitrogen transport, and overall catalytic selectivity.

### 3.3. Reactor Architectures for Electrochemical Ammonia Production

**Figure 4:**
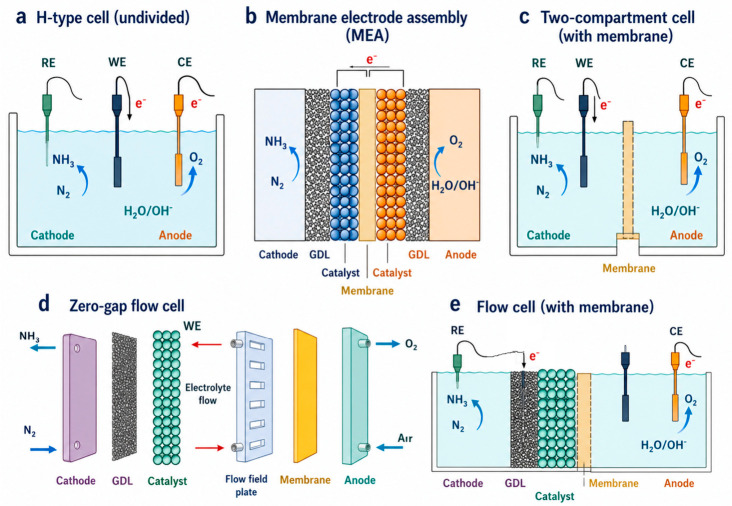
Schematic comparison of electrochemical reactor configurations for ammonia production. Abbreviations: GDL, gas diffusion layer; WE, working electrode; RE, reference electrode; CE, counter electrode.

The design of the electrochemical reactor is a determining factor in the practical performance of ammonia synthesis systems, as it directly controls reactant supply, interfacial contact, ionic transport, internal resistance, and product separation. Accordingly, reactor optimization must be considered together with catalyst and electrolyte design in order to achieve meaningful improvements in activity, selectivity, energy efficiency, and scalability. While early developments in this field primarily focused on catalyst optimization, recent studies have clearly demonstrated that reactor architecture is equally critical for overcoming limitations related to current density (CD), mass transport, and scalability. According to Zou et al. [22], electrochemical ammonia synthesis systems can be broadly grouped into batch liquid-phase reactors, continuous-flow configurations, and membrane electrode assembly (MEA)-based systems. These reactor types reflect a clear progression from simple laboratory-scale setups toward more integrated and scalable technologies, as schematically illustrated in Figure 4.

Batch liquid-phase reactors, including single-compartment and H-type cells, are still the most widely used platforms for catalyst evaluation and mechanistic studies. Single-compartment configurations are particularly appealing due to their simplicity and operational flexibility, and they have been extensively applied in lithium-mediated nitrogen reduction systems. However, the absence of a physical barrier between anodic and cathodic compartments leads to product crossover and parasitic reactions. Moreover, nitrogen availability is restricted by its low solubility in aqueous electrolytes (~0.7 mmol·L^−1^), limiting reactant transport to the electrode surface. Consequently, these systems typically operate at low current densities (<10–20 mA cm^−2^) and moderate Faradaic efficiencies. For instance, Andersen et al. [173] reported an FE of approximately 8% at atmospheric pressure, highlighting the impact of transport limitations and competing HER. Increasing system pressure can enhance nitrogen solubility, but does not fundamentally overcome interfacial transport constraints [171].

H-type reactors introduce a dual-compartment configuration separated by an ion-conducting membrane, allowing independent control of catholyte and anolyte conditions. This design reduces ammonia crossover and generally improves selectivity. Under optimized conditions, higher performance can be achieved. For example, Kong et al. [174] reported an ammonia production rate of 16.1 mg h^−1^ mg_cat_^−1^ with an FE of 11.8% in alkaline media. In specific cases, even higher FE values have been observed, such as 82.83% at −0.4 V vs. RHE using LiNb_3_O_8_ catalysts, although typically at relatively low current densities [175]. Despite these improvements, H-type cells remain limited by relatively high internal resistance and diffusion-controlled nitrogen transport, restricting their scalability and preventing operation under industrially relevant conditions. To address these limitations, continuous-flow reactor configurations have been developed to enhance mass transport and enable stable gas–liquid–solid interfaces. These systems are particularly advantageous for nitrogen reduction, where low N_2_ solubility constrains performance in conventional setups. A representative example is the three-chamber flow reactor developed by Wei et al. [159], which integrates a gas diffusion electrode with controlled electrolyte flow. This configuration achieved a Faradaic efficiency of 64.8%, an ammonia production rate of 9.9 × 10^−10^ mol cm^−2^ s^−1^, and an energy efficiency of 40.7% at −0.1 V vs. RHE, demonstrating the benefits of improved reactant delivery and interfacial control.

Flow reactor concepts have also been extended to alternative nitrogen sources. Hu et al. [176] reported a 16 cm^2^ flow electrolyzer coupling nitrate reduction with hydroxide evolution, achieving an ammonia production rate of 16.9 mmol h^−1^ at a cell voltage of 1.2 V and an energy consumption of 17 kWh kg^−1^ NH_3_, approaching industrial relevance. Similarly, a lithium-mediated continuous-flow system operating at −6 mA cm^−2^ achieved an FE of approximately 61% and an energy efficiency of around 13%, illustrating the trade-off between productivity and efficiency [177]. Despite these advances, challenges remain in terms of catalyst stability, local pH gradients, limited residence time, and increased system complexity.

Among the available architectures, MEA-based systems represent the most advanced approach from an engineering perspective. These systems employ a zero-gap configuration, where catalyst layers are directly interfaced with an ion-conducting membrane, minimizing ohmic losses and enabling operation at high current densities. For instance, a scalable MEA reactor achieved an FE of 91.8% at 500 mA cm^−2^, demonstrating that high selectivity can be maintained under industrially relevant conditions [178]. Similarly, a 5 cm^2^ MEA device operating via nitrate reduction delivered a partial ammonia current of 1.8 A with an FE of 91% [179].

Further improvements have been achieved through advanced membrane-based systems that integrate reaction and separation. A bipolar membrane continuous-flow reactor demonstrated stable operation at 1000 mA cm^−2^ for more than 100 h, achieving an FE of 86.2% and an ammonia production rate of 68.4 mg h^−1^ cm^−2^ in nitrate-containing electrolytes [180]. In parallel, flow-electrode configurations have reached nitrate conversion efficiencies of approximately 97% with ammonia recovery efficiencies close to 70%, highlighting the importance of process integration [181].

Reactor development has followed a clear evolution. Batch systems remain useful for fundamental investigations but are inherently limited by mass transport and resistance. Continuous-flow reactors improve interfacial transport and enable steady-state operation, while MEA-based configurations provide the highest performance by combining high current densities (up to 500–1000 mA cm^−2^), high Faradaic efficiencies (>85–90%), and improved scalability. Consequently, reactor architecture plays a central role in determining not only ammonia yield and selectivity, but also energy efficiency, long-term stability, and the feasibility of industrial deployment.

## 4. Techno-Economic and Sustainability Considerations in Electrochemical Ammonia Production

The performance constraints discussed in previous sections, particularly the low current density and Faradaic efficiency typically achieved in electrochemical systems, have a direct impact on the economic viability of ammonia production. Most experimental nitrogen reduction setups operate at CD values below 20 mA·cm^−2^ and FE below 35%, resulting in limited ammonia production rates and high energy consumption [182,183]. Techno-economic analyses suggest that, to compete with the conventional Haber–Bosch process, electrochemical systems would need to reach CD values above 300–400 mA·cm^−2^ and FE in the range of 30–90%, highlighting the substantial performance gap that remains [182,184].

At the system level, these limitations translate into elevated capital and operational costs. Electrolyzer units account for a large fraction of the capital expenditure, mainly due to the extensive electrode surface area required to compensate for low productivity. In some cases, the electrolyzer has been reported to represent up to approximately 90% of the total capital cost in nitrogen reduction-based systems [185]. In addition, low selectivity toward ammonia increases electricity consumption, with typical energy demands exceeding ~30 kWh·kg^−1^ NH_3_ [49]. These values remain significantly higher than those associated with conventional ammonia production, reinforcing the need for improvements in catalytic and system performance.

In contrast, nitrate reduction offers a more favourable techno-economic outlook due to improved thermodynamics and reaction kinetics. The lower bond dissociation energy of N–O bonds compared to the N≡N bond reduces the activation barrier, enabling higher reaction rates [24,186]. Experimental studies have reported CD values in the range of 1–2 A·cm^−2^ and FE up to approximately 95%, particularly when using transition metal catalysts such as Ni-based systems that effectively stabilise intermediates such as *NO_2_ and *NO [45,187]. These performance gains translate into smaller reactor sizes and reduced electricity consumption, with techno-economic models estimating ammonia production costs below $1 kg^−1^ NH_3_ under favourable conditions [185].

An additional advantage of nitrate reduction lies in its compatibility with alternative nitrogen sources. Wastewater streams containing nitrate concentrations up to approximately 2000 ppm can be used directly after appropriate pretreatment, thereby avoiding energy-intensive nitrogen separation steps [188]. Moreover, conventional nitrate removal processes typically involve costs in the range of $3–6 per kg NO_3_^−^, meaning that electrochemical conversion to ammonia may partially offset treatment costs [189]. However, the overall economic benefit is highly dependent on the nitrate source. When synthetic nitrate is employed, the cost of ammonia increases significantly due to the energy-intensive production of nitrate salts, as highlighted in environmental and techno-economic assessments [190].

Lithium-mediated nitrogen reduction represents another alternative that partially overcomes the kinetic limitations of direct N_2_ activation. This approach relies on the formation of lithium nitride (Li_3_N), enabling ammonia synthesis under ambient conditions [191]. Experimental studies have reported FE values up to approximately 70% and CD approaching 0.5–1 A·cm^−2^, representing a significant improvement compared to conventional aqueous nitrogen reduction systems [191,192]. More recent developments, including continuous-flow operation and improved electrode design, have further enhanced system productivity and stability [5,177]. However, these gains are accompanied by substantial energy penalties, as lithium deposition requires highly negative potentials, leading to cell voltages of approximately 3–4 V and relatively low overall energy efficiency [169,193].

The economic performance of lithium-mediated systems is also strongly influenced by hydrogen supply. Unlike aqueous systems, these processes typically rely on an external hydrogen source, which introduces additional operational costs [191,194]. Depending on how hydrogen is produced, this can significantly affect the overall cost structure. Furthermore, the use of organic electrolytes such as tetrahydrofuran introduces additional challenges related to system stability. Electrolyte degradation, solvent losses, and side reactions require periodic replacement, which increases operating costs and limits long-term scalability [189,190,192,193].

Ammonia separation remains another critical challenge for electrochemical processes. In aqueous systems, ammonia is often generated at concentrations below 1 wt%, particularly under laboratory conditions, making recovery energy-intensive [34]. At such low concentrations, distillation becomes inefficient, with energy demand increasing sharply as ammonia concentration decreases. Process simulations indicate that ammonia concentrations above approximately 10 wt% are required to achieve reasonable separation energy requirements [185]. In contrast, lithium-mediated systems predominantly produce ammonia in the gas phase, enabling alternative separation strategies such as adsorption, which can reduce energy demand compared to conventional distillation [195].

From an environmental perspective, electricity consumption is the dominant contributor to greenhouse gas emissions in electrochemical ammonia production. Reported energy requirements range from approximately 25 to 35 kWh·kg^−1^ NH_3_, depending on the reaction pathway and system performance [97]. Consequently, the carbon footprint is highly dependent on the electricity source. Under current grid electricity mixes, emissions may exceed those of conventional Haber–Bosch synthesis, whereas the use of low-carbon electricity can reduce emissions to below approximately 2–3 kg CO_2_ eq·kg^−1^ NH_3_, approaching sustainable production targets [97,196].

The origin of feedstocks also plays a key role in environmental performance. Using nitrate derived from wastewater can significantly reduce overall emissions by avoiding the environmental burden associated with nitrate discharge and treatment [189]. In contrast, synthetic nitrate production and fossil-derived hydrogen increase the carbon footprint due to upstream emissions [190]. Life cycle assessment studies consistently indicate that coupling electrochemical ammonia synthesis with renewable electricity and waste-derived nitrogen sources is essential to maximise environmental benefits [97].

The comparison of different electrochemical pathways reveals clear trade-offs between performance, cost, and sustainability. Nitrate reduction currently provides the most favourable combination of high CD, high FE, and lower energy demand, particularly when integrated with wastewater treatment systems [186,197]. Lithium-mediated nitrogen reduction offers improved selectivity and productivity but remains constrained by energy efficiency, hydrogen requirements, and electrolyte stability [192,194]. In contrast, direct nitrogen reduction continues to be limited by low reaction rates and high energy consumption, requiring substantial advances in catalyst development and system engineering to achieve economic viability [182,198].

## 5. Key Challenges and Future Directions in Electrochemical Ammonia Synthesis

Despite the significant advances achieved in recent years, electrochemical ammonia synthesis remains at an early stage of technological development and is not yet competitive with the conventional Haber–Bosch process. While electrochemical approaches offer clear advantages, such as operation under mild conditions and compatibility with renewable electricity, multiple limitations still hinder their practical implementation. These challenges span different scales, from fundamental reaction mechanisms to system integration and economic feasibility, and are often strongly interconnected.

One of the most persistent bottlenecks is the intrinsically low efficiency of the NRR. As discussed in previous sections, current systems are typically characterized by low Faradaic efficiency and limited ammonia production rates, mainly due to the difficulty of activating molecular nitrogen and the strong competition with side reactions, particularly hydrogen evolution. Addressing this limitation requires the development of catalysts capable of selectively promoting nitrogen activation while suppressing HER, a challenge that remains unresolved.

In this context, catalyst design continues to be a central focus of research. Although considerable progress has been achieved through strategies such as heteroatom doping, defect engineering, alloy formation, and the development of single-atom catalysts, existing materials still struggle to combine high activity, selectivity, and long-term stability. Future efforts should therefore move beyond simply increasing the density of active sites and instead focus on tailoring their electronic structure to enable efficient adsorption and controlled hydrogenation of intermediates. In parallel, catalyst durability must be systematically addressed, as many high-performing materials undergo deactivation, surface restructuring, or poisoning during extended operation. The integration of data-driven methodologies, including machine learning and multiscale modelling, is expected to accelerate catalyst discovery by identifying structure–property relationships and guiding the design of optimized materials.

A further challenge lies in the limited mechanistic understanding of electrochemical ammonia synthesis. Reaction pathways are highly dependent on catalyst composition, electrolyte properties, and local interfacial conditions, making it difficult to establish general design rules. Although theoretical studies have provided valuable insights, discrepancies between computational predictions and experimental observations are still common due to the complexity of real electrochemical environments. To bridge this gap, future work should increasingly rely on in situ and operando characterization techniques, such as Raman spectroscopy, infrared spectroscopy, and electrochemical mass spectrometry, which enable direct observation of intermediates and catalyst evolution under working conditions. Combining these experimental approaches with advanced modelling will be essential to translate fundamental insights into practical catalyst design.

Electrolyte engineering also remains a critical area for further development. As highlighted previously, the electrolyte plays a key role in determining proton availability, interfacial charge distribution, nitrogen solubility, and intermediate stabilization. However, current electrolyte systems often involve trade-offs between conductivity and selectivity. In aqueous media, high proton concentrations promote HER, whereas strategies to suppress HER, such as reducing water activity or introducing additives, can negatively affect ionic transport. Future research should therefore aim to decouple these effects through advanced electrolyte design, including confined electrolytes, molecular crowding approaches, and tailored ion–solvent interactions. In nonaqueous and lithium-mediated systems, additional challenges arise from electrolyte instability, sensitivity to impurities, and the formation of complex interphases that govern reaction kinetics. Developing stable and scalable electrolyte systems will be essential for long-term operation.

From an engineering perspective, reactor design and mass transport limitations continue to restrict system performance. In particular, the low solubility of nitrogen in aqueous electrolytes severely limits its availability at the catalyst surface, thereby constraining achievable current densities. Although gas diffusion electrodes and flow-cell configurations have demonstrated significant improvements, many studies still rely on H-type reactors that are not representative of practical applications. Future progress will require the development of advanced reactor architectures, including continuous-flow systems and MEA configurations, capable of operating at high current densities with efficient reactant delivery and reduced energy losses. At the same time, issues such as membrane durability, product crossover (e.g., NH_4_^+^ transport), and long-term operational stability must be addressed to enable scale-up.

In the case of electrochemical nitrate reduction, which currently exhibits more favourable performance metrics, the main challenge shifts toward achieving high selectivity within a complex reaction network. The reduction of nitrate involves multiple intermediates and competing pathways leading to products such as NO_2_^−^, NO, N_2_O, and N_2_. Maximizing ammonia selectivity therefore requires precise control over catalyst properties, hydrogen availability, and local reaction conditions. In addition, bridging the gap between mechanistic understanding and practical implementation remains a key issue. While theoretical studies have clarified many aspects of the reaction mechanism, their translation into scalable systems is still limited. Future research should focus on integrating mechanistic insights into catalyst and reactor design, as well as validating performance under realistic conditions, including wastewater matrices. These challenges illustrate that future progress will depend on bridging fundamental mechanistic understanding with integrated system-level optimization under realistic operating conditions.

Another critical issue across all electrochemical ammonia synthesis routes is the reliability of ammonia detection and quantification. Due to the typically low production rates, even trace contamination from experimental setups can lead to significant overestimation of catalytic performance. This has contributed to inconsistencies in the literature and, in some cases, questionable claims regarding catalyst activity. The adoption of rigorous validation protocols, including isotopic labeling techniques (e.g., ^15^N-based methods), control experiments, and standardized analytical procedures, is therefore essential to ensure reproducibility and comparability of results.

Beyond technical challenges, economic viability and scalability remain decisive for future deployment. Current electrochemical systems generally exhibit higher energy consumption and lower productivity than the Haber–Bosch process, resulting in higher costs. Achieving industrial relevance will require simultaneous improvements in current density, Faradaic efficiency, catalyst lifetime, and reactor design. In addition, system integration strategies, such as coupling ammonia synthesis with renewable electricity sources, wastewater treatment, or value-added anodic reactions, may enhance overall process economics. In particular, nitrate reduction offers a promising near-term pathway due to its dual function of pollutant removal and ammonia production, provided that energy consumption and system complexity can be minimized.

Looking ahead, further progress in electrochemical ammonia synthesis will likely depend on a transition from isolated material-level studies toward fully integrated system design. Advances in catalyst development, electrolyte engineering, and reactor configuration must be pursued in a coordinated manner to achieve meaningful performance improvements. At the same time, emerging tools such as machine learning, high-throughput experimentation, and operando characterization are expected to accelerate innovation and provide deeper insight into reaction mechanisms. In particular, data-driven materials innovation strategies are increasingly being used to identify structure–property relationships and guide catalyst discovery [199]. In parallel, robotic and multimodal autonomous experimentation platforms have recently demonstrated the capability to accelerate the synthesis, screening, and optimization of advanced catalytic materials through the integration of artificial intelligence, automated experimentation, and real-time data analysis [200,201]. These approaches are expected to ultimately support the development of efficient and scalable ammonia production technologies.

## 6. Conclusions

Electrochemical routes for ammonia production have progressed notably in recent years, but they are still far from reaching industrial relevance. The discussion presented here highlights that the main bottlenecks are not linked to a single component, but rather to the intrinsic complexity of the overall system and the need to optimize catalysts, electrolytes, and reactor design in a coordinated manner. Among the different pathways, nitrate reduction currently stands out due to its higher reaction rates and efficiencies, whereas direct nitrogen reduction remains constrained by fundamental kinetic and thermodynamic limitations. Future advances will therefore require closing the gap between mechanistic insights and practical implementation, as well as demonstrating stable operation and competitive energy performance under realistic conditions. Achieving this level of integration will be essential for transforming electrochemical ammonia synthesis from a laboratory-scale concept into a technically and economically viable alternative.

## Figures and Tables

**Table 1:** Comparative performance of representative electrocatalysts for electrochemical nitrogen reduction reaction toward ammonia synthesis.

Catalyst	Electrolyte	NH_3_ Yield	FE (%)	Potential (V vs. RHE)	Reference
**Noble metal-based catalysts**					
Au/o-CFP	0.1 M Na_2_SO_4_	40.6 μg h^−1^ mg_cat_^−1^	31.3	−0.3 (yield); −0.1 (FE)	[65]
Au_1_Cu_1_	0.05 M H_2_SO_4_	154.91 μg h^−1^ mg_cat_^−1^	54.96	−0.2	[66]
Au_3_Cu@Cu nanocages	0.1 M Na_2_SO_4_	33.97 μg h^−1^ mg_cat_^−1^	21.41	−0.2	[67]
Au@TiO_2_	0.1 M HCl	64.6 μg h^−1^ mg_cat_^−1^	29.5	—	[68]
HT Au@MOF	0.1 M Na_2_SO_4_	49.5 μg h^−1^ mg_cat_^−1^	60.9	−0.3	[69]
B,S-mRh/NF	0.1 M Na_2_SO_4_	11.88 μg h^−1^ cm_cat_^−2^	38.42	−0.1 (yield); −0.05 (FE)	[70]
Ru-TiNS	0.1 M KHCO_3_	15.19 μmol h^−1^ mg_cat_^−1^	15.3	−0.3	[71]
Ru nano-based/rGO	0.1 M H_2_SO_4_	9.14 μg h^−1^ mg_cat_^−1^	2.1	−0.2	[72]
Ru_2_P-rGO	0.1 M HCl	32.8 μg h^−1^ mg_cat_^−1^	13.04	−0.05	[73]
BCC PdCu	LiCl	35.7 μg h^−1^ mg_cat_^−1^	11.5	−0.1	[74]
PdH0.43 NRs	0.1 M Na_2_SO_4_	17.53 μg h^−1^ mg_cat_^−1^	18.56	0.2	[75]
PdP_2_-rGO	0.5 M LiClO_4_	30.3 μg h^−1^ mg_cat_^−1^	12.56	−0.1	[76]
Pt SAs/WO_3_	0.1 M K_2_SO_4_	342.4 μg h^−1^ mg^−1^ Pt	31.1	—	[77]
Pd/γ-MnO_2_	0.1 M KOH	19.72 μg h^−1^ mg^−1^ Pd	8.4	—	[78]
Pd0.2Cu0.8/rGO	0.1 M KOH	2.8 μg h^−1^ mg_cat_^−1^	~4.5	—	[79]
PdO/Pd/CNTs	0.1 M NaOH	18.2 μg h^−1^ mg_cat_^−1^	11.5	—	[80]
**Non-noble metal-based catalysts**					
Fe (after activation)	0.01 M PBS	26.44 μg h^−1^ mg_cat_^−1^	20.4	—	[81]
Fe/TiO_2_	0.5 M LiClO_4_	25.47 μg h^−1^ mg_cat_^−1^	25.6	—	[82]
Fe-doped Bi_2_MoO_6_	0.1 M HCl	71.01 μg h^−1^ mg_cat_^−1^	80.12	—	[83]
Fe–N/C-CNTs	0.1 M KOH	34.83 μg h^−1^ mg_cat_^−1^	9.28	−0.3	[84]
FeS@MoS_2_/CFC	0.1 M KOH	2.46 μg h^−1^ mg_cat_^−1^	0.067	−0.15 (yield); −0.1 (FE)	[85]
LaxFeO_3_−δ	0.1 M Li_2_SO_4_	22.1 μg h^−1^ mg_cat_^−1^	25.6	—	[86]
MoS_2_/MoO_2_@Fe_2_O_3_/BC	0.1 M Na_2_SO_4_	4.87 μg h^−1^ cm_cat_^−2^	16.5	−0.8	[87]
Mo_2_C-MoO_2_@RGO	0.1 M Na_2_SO_4_	13.94 ± 0.39 μg h^−1^ mg_cat_^−1^	12.72 ± 0.58	−0.5 (yield); −0.1 (FE)	[88]
SA-Mo/NPC	1.0 M KOH	34 μg h^−1^ mg_cat_^−1^	14.6	—	[89]
Mo-SnO_2_/C	0.1 M Na_2_SO_4_	24.03 μg h^−1^ mg_cat_^−1^	7.11	—	[90]
Ni^3+^/MoO_2_	0.1 M Na_2_SO_4_	10.6 μg h^−1^ mg_cat_^−1^	19.6	−0.4	[91]
Defect-rich γ-Mo_2_N/h-BN	0.1 M Na_2_SO_4_	58.5 μg h^−1^ mg_cat_^−1^	61.5	−0.7	[92]
np-OVs NiO/MoO_3_	0.1 M PBS	35.4 μg h^−1^ mg_cat_^−1^	10.3	—	[93]
oxGR-NS	PBS	28.5 μg h^−1^ mg_cat_^−1^	13.2	—	[94]
H-NiCo-NC	0.1 M Na_2_SO_4_	52.8 μg h^−1^ mg_cat_^−1^	11.5	—	[95]
Zn-doped Co_3_O_4_	0.1 M HCl	22.71 μg h^−1^ mg_cat_^−1^	11.9	—	[96]
Mn–N–C SAC	0.1 M HCl	21.43 μg h^−1^ mg_cat_^−1^	32.02	—	[97]
K_3_Ti_8_O_17_	0.1 M HCl	31.6 μg h^−1^ mg_cat_^−1^	15	—	[98]
Nb_2_O_5_-NCF	0.1 M Na_2_SO_4_	2.52 × 10^−10^ mol cm^−2^ s^−1^	9.81	—	[97]
Ti_2_NTx MXene	0.1 M HCl	11.33 μg h^−1^ cm^−2^	19.85	—	[44]
MnO_2_-Ti_3_C_2_Tx	0.1 M HCl	34.12 μg h^−1^ mg_cat_^−1^	11.39	—	[99]
CoP hollow nanocage	1.0 M KOH	10.78 μg h^−1^ mg_cat_^−1^	7.36	—	[100]
**Metal-free catalysts**					
BNQDs/C_3_N_4_	0.5 M LiClO_4_	72.3 μg h^−1^ mg_cat_^−1^	19.5	−0.3 (yield); −0.2 (FE)	[101]
F-doped carbon	0.05 M H_2_SO_4_	197.7 μg h^−1^ mg_cat_^−1^	54.8	−0.3 (yield); −0.2 (FE)	[102]
Cl-RGO	0.05 M H_2_SO_4_	70.9 μg h^−1^ mg_cat_^−1^	5.97	−0.3	[103]
SDG	0.5 M LiClO_4_	28.56 μg h^−1^ mg_cat_^−1^	7.07	−0.85	[104]
NPC-750	0.05 M H_2_SO_4_	1.40 mmol g^−1^ h^−1^	1.42	−0.9	[105]
BG-1	0.05 M H_2_SO_4_	9.8 μg h^−1^ cm_cat_^−2^	10.8	−0.5	[106]
B-BCN	0.05 M Na_2_SO_4_	8.39 μg h^−1^ cm_cat_^−2^	9.87	−0.3 (yield); −0.6 (FE)	[107]
M-COFBTC	0.05 M HCl	1.56 × 10^−11^ mol cm^−2^ s^−1^	4.51	—	[108]
Eex-COF/NC	0.1 M KOH	12.53 μg h^−1^ mg^−1^	45.43	—	[109]
N-doped nanoporous Carbon Membranes (NCM)	0.1 M HCl	0.08 g m^−2^ h^−1^	5.2	—	[110]
S-NV-C_3_N_4_	0.5 M LiClO_4_	32.7 μg h^−1^ mg_cat_^−1^	14.1	—	[111]
FL-BP NSs	0.01 M HCl	31.37 μg h^−1^ mg_cat_^−1^	5.07	—	[112]
B/N-CNF	0.1 M KOH	20.0 μg h^−1^ mg_cat_^−1^	13.2	—	[99]

**Table 2:** Comparative performance of representative electrocatalysts for electrochemical nitrate reduction reaction toward ammonia synthesis.

Catalyst	Electrolyte	NH_3_ Yield	FE (%)	Potential (V vs. RHE)	Reference
**Noble metal-based catalysts**					
Ag/ZnO	1 M KOH + NO_3_^−^	516 mmol g_approx_^−1^ h^−1^	66	−0.6	[113]
Ag@TiO_2_/TP	0.1 M NaOH + 0.1 M NO_2_^−^	514.3 mmol h^−1^ cm^−2^	96.4	−0.5	[114]
Ag_1.5_Co/CC	0.5 M K_2_SO_4_ + 200 ppm NO_3_^−^	0.227 mmol h^−1^ cm^−2^	96.11	−0.65	[115]
Au/Cu_2_O NCs	0.5 M K_2_SO_4_ + 0.05 M NO_3_^−^	39.79 mg h^−1^ mg_cat_^−1^	92.93	−0.75	[116]
Bi_1_Pd	1 M KOH + 0.1 M NO_3_^−^	33.8 mg h^−1^ cm^−2^	~100	−0.6	[117]
Cu-Pd/C	0.1 M KOH + 10 mM KNO_3_	220.8 mg mg_cat_^−1^ h^−1^	62.3	−0.4	[118]
Pd/NF	0.5 M Na_2_SO_4_ + 0.1 M NaNO_3_	1.52 mmol cm^−2^ h^−1^	78	−1.4	[119]
Pd_1_/BN	0.5 M Na_2_SO_4_ + 0.1 M NaNO_2_	347.1 mmol h^−1^ cm^−2^	91.7	−0.6	[120]
PdCu	0.5 M K_2_SO_4_ + 0.1 M KNO_3_	295 mg h^−1^ mg_cat_^−1^	90.9	−0.8	[121]
Pd_74_Ru_26_	1 M KOH + 100 mM NO_3_^−^	16.20 mg h^−1^ cm^−2^	Nearly 100	−0.3	[122]
Ru-25CV/oNF300	1 M KOH + 1 M NaNO_3_	3.6 × 10^−7^ mol s^−1^ cm^−2^	Nearly 100	−0.1	[123]
Ru@C_3_N_4_/Cu	0.5 M Na_2_SO_4_ + 200 ppm NO_3_^−^	0.249 mmol h^−1^ cm^−2^	91.3	−0.8	[124]
Ru NCs/TiO_2_ NTs	0.05 M Na_2_SO_4_ + 100 ppm NO_3_^−^	601 mg h^−1^ cm^−2^	>90.0	−0.4	[125]
Ru/O-doped-Ru core/shell	1 M KOH + 1 M KNO_3_	5.56 mol g_cat_^−1^ h^−1^	Nearly 100	−0.2	[126]
Ru_1_Cu_10_/rGO	1 M KOH + 0.1 M KNO_3_	0.38 mmol cm^−2^ h^−1^	98	−0.05	[127]
Ru/b-Co(OH)_2_	1 M KOH + 0.1 M KNO_3_	1.15 mmol cm^−2^ h^−1^	98.4	0.071	[128]
RuO_x_/Pd	1 M KOH + 0.1 M KNO_3_	23.5 mg h^−1^ cm^−2^	98.6	−0.5	[129]
**Non-noble metal-based catalysts**					
A_5_Fe_2_O_4_	1 M KOH + 0.1 M KNO_3_	2.1 mmol h^−1^ cm^−2^	98.1	−0.5	[130]
B/Fe-Co_2_P HNCs	0.5 M Na_2_SO_4_ + 50 mM NaNO_3_	22.67 mg h^−1^ mg_cat_^−1^	97.54	−0.7	[131]

## Data Availability

No new data were created or analyzed in this study. Data sharing is not applicable to this article.

## Abbreviations

The following abbreviations are used in this manuscript:

CD	Current density
DFT	Density functional theory
FE	Faradaic efficiency
HER	Hydrogen evolution reaction
LiMEAS	Lithium-mediated electrochemical ammonia synthesis
MEA	Membrane electrode assembly
NRR	Nitrogen reduction reaction
PBS	Phosphate-buffered saline
RHE	Reversible hydrogen electrode
rGO	Reduced graphene oxide
